# Ternary Heterojunction Synaptic Transistors Based on Perovskite Quantum Dots

**DOI:** 10.3390/nano15090688

**Published:** 2025-05-01

**Authors:** Shuqiong Lan, Jinkui Si, Wangying Xu, Lan Yang, Jierui Lin, Chen Wu

**Affiliations:** Department of Physics, School of Science, Jimei University, Xiamen 361021, China; 202412854025@jmu.edu.cn (J.S.); wyxu@jmu.edu.cn (W.X.); tiger0548@sina.com (L.Y.); 202312854020@jmu.edu.cn (J.L.); 202412854012@jmu.edu.cn (C.W.)

**Keywords:** ternary heterojunction, synaptic transistors, perovskite quantum dots, synergistic trapping

## Abstract

The traditional von Neumann architecture encounters significant limitations in computational efficiency and energy consumption, driving the development of neuromorphic devices. The optoelectronic synaptic device serves as a fundamental hardware foundation for the realization of neuromorphic computing and plays a pivotal role in the development of neuromorphic chips. This study develops a ternary heterojunction synaptic transistor based on perovskite quantum dots to tackle the critical challenge of synaptic weight modulation in organic synaptic devices. Compared to binary heterojunction synaptic transistor, the ternary heterojunction synaptic transistor achieves an enhanced hysteresis window due to the synergistic charge-trapping effects of acceptor material and perovskite quantum dots. The memory window decreases with increasing source-drain voltage (V_DS_) but expands with prolonged program/erase time, demonstrating effective carrier trapping modulation. Furthermore, the device successfully emulates typical photonic synaptic behaviors, including excitatory postsynaptic currents (EPSCs), paired-pulse facilitation (PPF), and the transition from short-term plasticity (STP) to long-term plasticity (LTP). This work provides a simplified strategy for high-performance optoelectronic synaptic transistors, showcasing significant potential for neuromorphic computing and adaptive intelligent systems.

## 1. Introduction

The traditional von Neumann computing architecture suffers from low computational efficiency and high power consumption due to the separation of memory and computation units, which fails to meet the demands for information processing in the era of big data [1,2,3,4]. Inspired by the human brain, optoelectronic neuromorphic computing offers advantages such as ultra-high speed, large bandwidth, multidimensional capabilities, and low power consumption. It holds significant potential for applications in high-performance computing and artificial intelligence [5,6,7,8,9]. The optoelectronic synaptic device serves as a fundamental hardware foundation for realizing neuromorphic computing and is also key to the development of neuromorphic chips [10].

The phenomenon in which the strength of connections between neurons can increase or decrease in response to variations in their own neural activity is known to as synaptic plasticity [11]. The most fundamental and significant characteristic of synapses is adjustable synaptic plasticity. This adaptability corresponds to various biological functions of synapses and is crucial for memory and learning capabilities. Currently, artificial optoelectronic synaptic devices primarily consist of two-terminal memristors and three-terminal synaptic transistors [12,13,14,15]. Among these, three-terminal synaptic transistors offer advantages such as superior stability, controllable testing parameters, and the ability to enable parallel learning, making them more conducive to achieving advanced synaptic functions. Furthermore, by adjusting the gate voltage, the channel conductance can be progressively modulated, which closely resembles biological synaptic behavior [16,17,18]. Various materials, including organic semiconductors, metal oxides, and 2D materials, have been successfully employed in optoelectronic synaptic transistors [19,20,21,22]. Notably, organic semiconductors emerge as ideal channel materials for high-performance photoelectronic synaptic transistors due to their advantages of a low cost, ease of processing, biocompatibility, and flexibility [23,24,25]. However, organic synaptic transistors still face significant challenges in effective synaptic weight modulation.

The modulation of synaptic plasticity in organic synaptic transistors is typically achieved by introducing additional functional layers. For example, Wang et al. incorporated a two-dimensional layer (MoS_2_) onto the organic semiconductor (PTCDA) to effectively regulate physical processes such as carrier trapping and release, thereby modulating the synaptic plasticity of the device and successfully emulating typical optoelectronic synaptic behaviors [26]. Gao et al. utilized IDTBT as the channel material and PVA as the capture layer, while integrating a bilayer heterojunction (POFDID/N2200) between PVA and SiO_2_ to fabricate organic synaptic transistors. The photogenerated carriers from the heterojunction were separated and trapped by the heterojunction barrier, creating a spatial electric field that screened the gate voltage. This process enabled the precise modulation of carrier capture and release, thus achieving mixed-weight synaptic plasticity [27]. However, the addition of functional layers or extra charge capture regulation layers increased both the complexity and cost of device fabrication.

Bulk heterojunctions utilize a blend of donor and acceptor materials, offering simple structures and excellent photosensitivity, which have been investigated for application in synaptic transistors [28,29,30]. However, the limited carrier capture capability of a single acceptor constrains the synaptic plasticity of devices, highlighting an urgent need for novel strategies to enhance this property. Perovskite quantum dots have garnered significant attention due to their remarkable properties of high carrier mobility, solution processability, tunable band structures, and strong light absorption. These properties present significant potential for applications in optoelectronic synaptic devices [31,32].

Therefore, this work develops ternary bulk heterojunction synaptic transistors by doping donor materials with a small amount of acceptor material (PC_61_BM) and perovskite quantum dots (CsPbBr_3_ QDs). In this design, both the acceptor material and perovskite quantum dots serve as charge trapping centers, synergistically enhancing carrier trapping efficiency while simplifying the device architecture. In comparison to binary heterojunction synaptic transistors, the ternary heterojunction synaptic transistor exhibits an enhanced hysteresis window due to the synergistic charge-trapping effects of PC_61_BM and CsPbBr_3_ QDs. The memory window decreases with increasing source-drain voltages (V_DS_) yet expands with prolonged program/erase time, thereby demonstrating effective modulation of carrier trapping. Furthermore, this study successfully emulates typical synaptic behaviors, such as excitatory postsynaptic currents (EPSCs) under varying light intensities or light pulse widths, paired-pulse facilitation (PPF), and the transition from short-term plasticity (STP) to long-term plasticity (LTP), accompanied by a thorough mechanistic interpretation. This work demonstrates significant potential for applications in neuromorphic computing and optoelectronic intelligent systems.

## 2. Materials and Methods

PDVT-10 (Mw > 50,000, PDI < 3) was purchased from Derthon Optoelectronic Materials Technology Co., Ltd., (Shenzhen, China) and CsPbBr_3_ quantum dot solution was obtained from Xianfeng Nanomaterials Technology Co., Ltd. PDVT-10 (5 mg) and PC_61_BM (5 mg) were separately dissolved in chloroform (1 mL) and then heated on a heated platform at 60 °C for 8 h. Subsequently, the PDVT-10, PC_61_BM, and CsPbBr_3_ QDs were mixed at a concentration ratio of 70:15:15 to form a ternary blend solution.

The Si wafer containing a 100 nm SiO_2_ layer was utilized as the substrate, where the SiO_2_ and Si served as the insulating layer and gate electrode of the synaptic transistor, respectively. First, the Si wafer was sequentially cleaned with acetone, isopropanol, and deionized water, followed by drying under nitrogen. The cleaned substrate was then treated with octadecyltrichlorosilane (OTS) for 20 min. Subsequently, the ternary blend solution was spin-coated onto the OTS-treated substrate at 1000 rpm for 60 s and then annealed at 90 °C for 30 mi. After depositing the ternary bulk heterojunction, 50 nm of Au was thermally evaporated onto the channel layer (channel length = 30 μm; channel width = 1000 μm) through a shadow mask to form source and drain electrodes.

All measurements were conducted under ambient conditions at room temperature. The electrical characteristics and synaptic behaviors were investigated using a semiconductor parameter analyzer (Keysight B2912B). Atomic Force Microscopy (AFM) (Bruker Dimension Icon) was employed to characterize the morphology of the ternary heterojunction. A UV-Vis spectrophotometer (Shimadzu UV-2600i) was utilized to measure the absorption spectrum of the thin films. 

## 3. Results and Discussion

**Figure 1:**
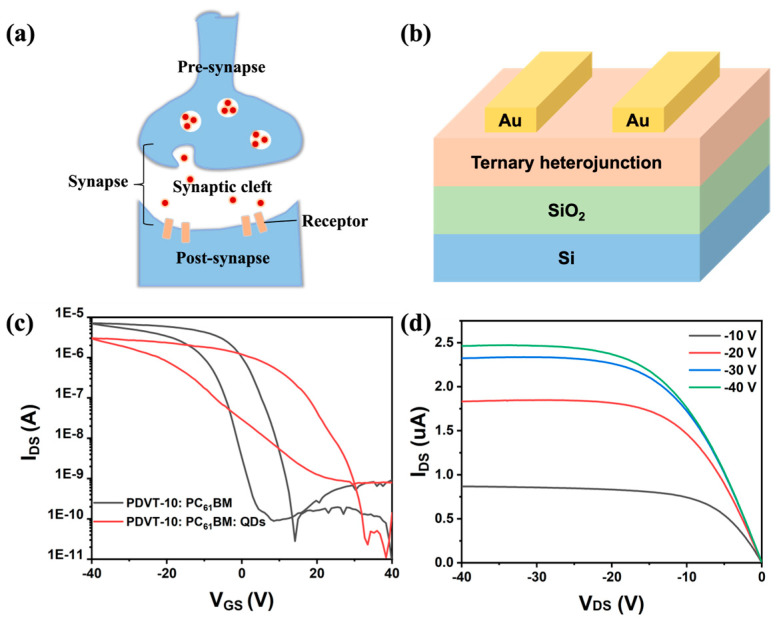
(**a**) Schematic diagram of biological synapse. (**b**) Device structure of ternary heterojunction synaptic transistor. (**c**) Comparison of dual-sweep transfer characteristics (V_DS_ = −10 V) of binary heterojunction synaptic transistor and ternary heterojunction synaptic transistor. (**d**) Output characteristic curves of ternary heterojunction synaptic transistor.

A schematic diagram of a biological synapse is depicted in Figure 1a, which primarily consists of the presynaptic neuron, postsynaptic neuron, synaptic cleft, and neurotransmitters. Signals originating from the presynaptic neuron stimulate the release of neurotransmitters, thereby effectively transmitting information to the postsynaptic neuron. Figure 1b illustrates the device architecture of the ternary heterojunction synaptic transistor developed in this study. The dual-sweep transfer curves of the binary heterojunction synaptic transistor and ternary heterojunction synaptic transistor were measured. As shown in Figure 1c, the hysteresis window of the ternary heterojunction synaptic transistor was significantly larger than that of the binary counterpart. This enhancement can be attributed to the incorporation of perovskite quantum dots, which increases the number of charge carrier trapping centers and thereby improves both the charge carrier capture and release capabilities in the ternary heterojunction synaptic transistor. Such superior charge-trapping dynamics endows the ternary heterojunction device with promising potential for high-efficiency neuromorphic computing systems. The output curves under different gate voltages are presented in Figure 1d. At low voltage, the curves exhibit a linear trend, indicating that the ternary heterojunction film had good contact with the Au electrode.

**Figure 2:**
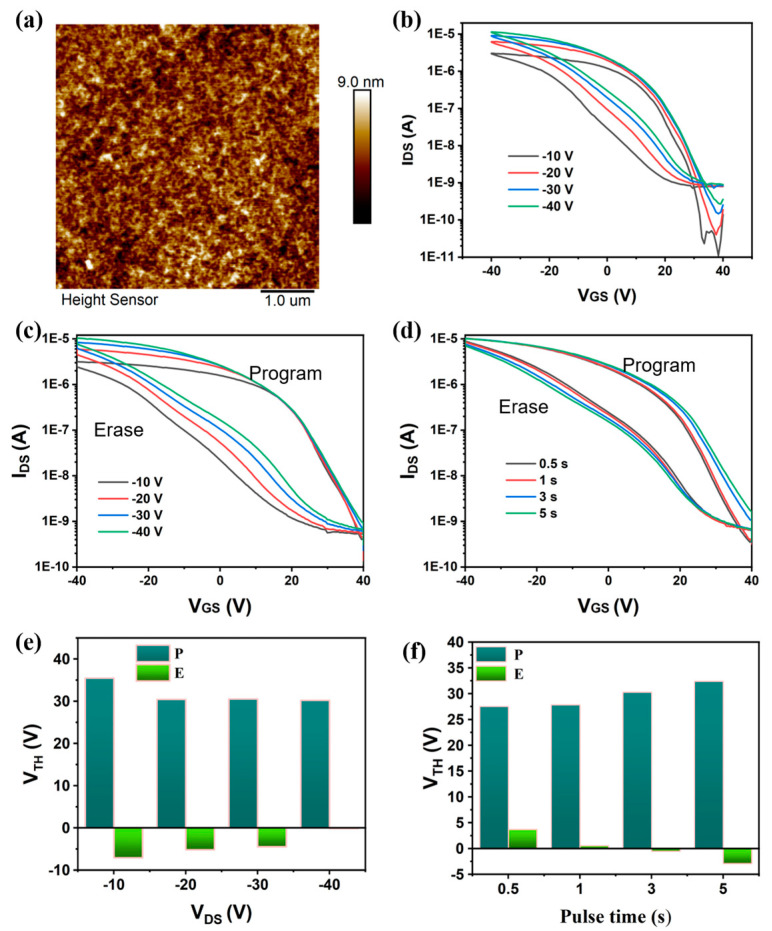
(**a**) An AFM image of the ternary heterojunction film. (**b**) Dual-sweep transfer characteristic curves of the synaptic transistor based on the ternary heterojunction under different V_DS_. (**c**) The effect of different V_DS_ on the memory window (program/erase time = 3 s). (**d**) The effect of different program/erase times on the memory window (V_DS_ = −40 V). (**e**) The variation in V_TH_ under different V_DS_. (**f**) The variation in V_TH_ with respect to varying program/erase times.

The AFM image of the ternary heterostructure is shown in Figure 2a. It exhibits a uniform distribution of nano-scale morphological features, with a root mean square (RMS) roughness of only 1.32 nm. This smooth surface morphology effectively suppressed the formation of defects at both the channel layer/insulator and channel layer/Au electrode interfaces. The transfer characteristic curves under different source-drain voltages are presented in Figure 2b. As V_DS_ increased, enhanced carrier mobility in the channel shortened the interaction time between carriers and trapping centers (PC_61_BM and CsPbBr_3_ QDs), thereby reducing carrier trapping probability and ultimately mitigating the hysteresis window.

To investigate the carrier trapping modulation capability of ternary heterojunction synaptic transistors, we systematically studied the effects of different V_DS_ as well as program/erase times on the memory window. As shown in Figure 2c, when V_DS_ increased from −10 V to −40 V (program voltage = 40 V; erase voltage = −40 V; program/erase time = 3 s), the memory window of the ternary heterojunction synaptic transistor decreased from 42.5 V to 30.4 V, which aligned with the dual-sweep transfer characteristics. Figure 2d demonstrates that as the program/erase time increased from 0.5 s to 5 s (V_DS_ = −40 V; program voltage = 40 V; erase voltage = −40 V), the memory window expanded from 31.2 V to 35.3 V. The trapped carrier density (ΔN) can be estimated using the following equation [30]:

(1) $$\Delta N=\frac{C_{i}∆V_{TH}}{e}$$

where *C*_*i*_ represents the capacitance per unit area of the dielectric layer, e is the elementary charge, and ∆*V*_*TH*_ is the threshold voltage shift. An enlarged memory window indicates enhanced charge trapping density. Therefore, increasing the program/erase time can effectively modulate the number of trapped carriers.

**Figure 3:**
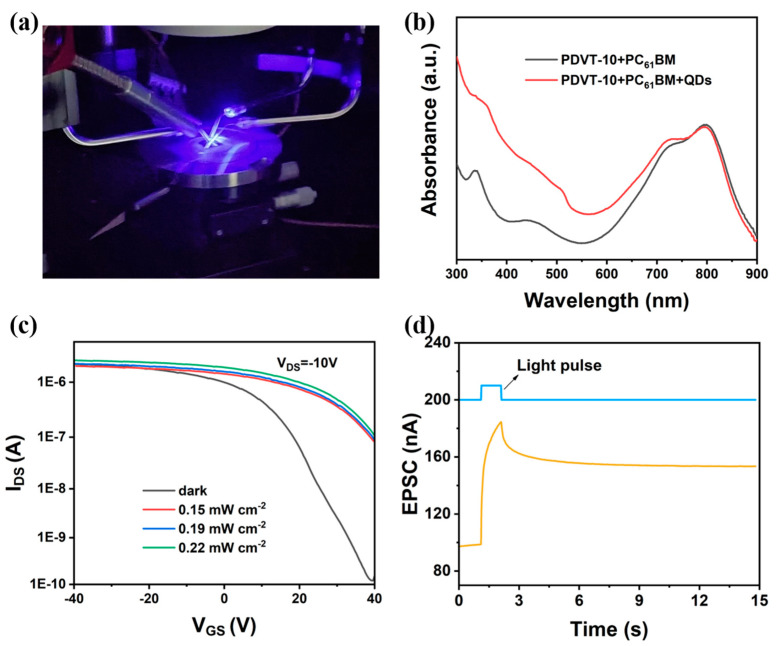
(**a**) Photograph of synaptic performance measurement. (**b**) Absorption spectra of binary heterojunction, PDVT-10–PC_61_BM, and ternary heterojunction, PDVT-10–PC_61_BM–CsPbBr_3_ QDs, thin films. (**c**) Transfer characteristics curves of synaptic transistor based on ternary heterojunction under different light intensities at 450 nm. (**d**) EPSC response of ternary heterojunction synaptic transistor under 450 nm light pulse (light intensity of 0.22 mW/cm^2^, pulse width of 1000 ms).

Figure 3a presents a photograph illustrating the measurement of synaptic performance. The absorption spectra of the binary heterojunction (PDVT-10–PC_61_BM) film and ternary heterojunction (PDVT-10–PC_61_BM–CsPbBr_3_ QDs) film are depicted in Figure 3b. Compared with the binary heterojunction films, the ternary heterojunction films enhanced the light absorption in the near-UV region. Figure 3c illustrates the transfer characteristic curves of the ternary heterojunction synaptic transistor under varying light intensities at 450 nm. Under illumination, the ternary heterojunction film generated excitons through photon absorption, which subsequently dissociated into free electrons and holes under the action of the interface barrier between PDVT-10, CsPbBr_3_ QDs, and PC_61_BM. Free electrons were trapped by CsPbBr_3_ QDs and PC_61_BM, while the hole concentration in the channel increased significantly. Consequently, the photoresponsive current was enhanced, accompanied by a pronounced positive threshold voltage shift in the transfer curves. Figure 3d shows the EPSC response of the ternary heterojunction synaptic transistor, where a 450 nm light pulse (light intensity = 0.22 mW/cm^2^; pulse width = 1000 ms) served as the pre-synaptic input signal and the source-drain current acted as the postsynaptic output signal (V_DS_ = −10 V). Upon illumination, the EPSC rapidly rose to 184.5 nA, followed by a gradual decay after light pulse termination.

**Figure 4:**
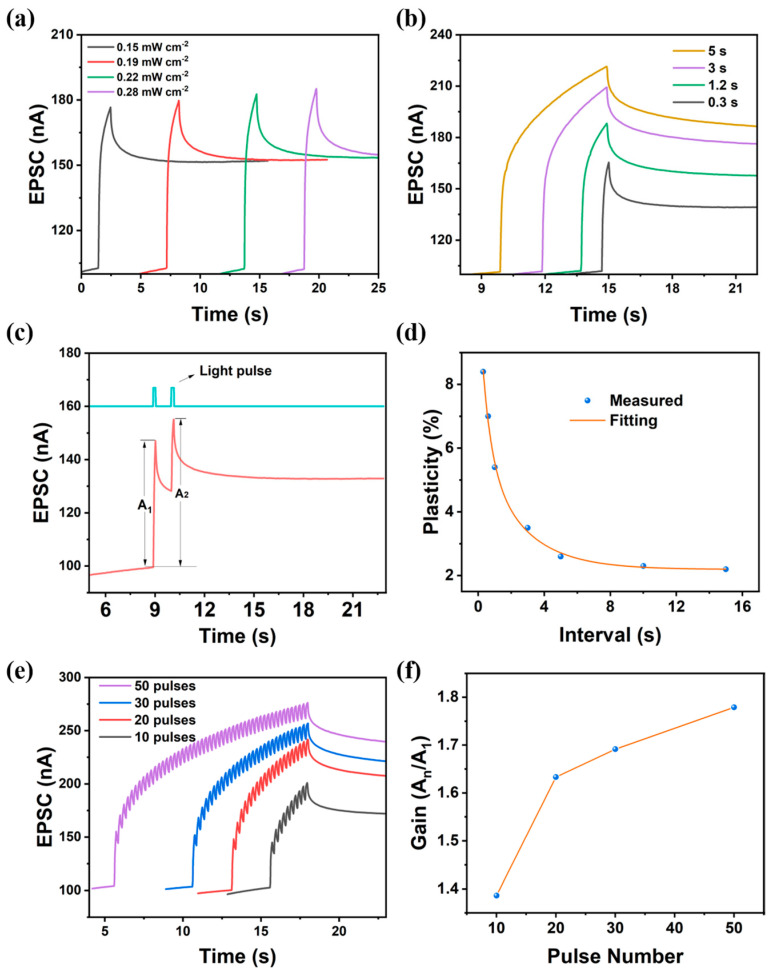
(**a**) Variation in EPSC with respect to light pulse intensity. (**b**) Variation in EPSC with respect to light pulse width. (**c**) Simulation of PPF (pulse width = 0.1 s; pulse interval = 1 s). (**d**) PPF fitted with double exponential function. (**e**) Evolution of EPSC under sequential light pulse stimulation with varying pulse numbers (pulse width = 0.1 s; pulse interval = 0.15 s). (**f**) Variation in gain (A_n_/A_1_) as function of pulse number.

The EPSC of the ternary heterojunction could be modulated by controlling light pulse intensity and duration. As shown in Figure 4a, under a fixed light pulse width (1000 ms), the EPSC peak amplitude was enhanced when light intensity increased from 0.15 mW/cm^2^ to 0.28 mW/cm^2^. This weak intensity dependence originated from the limited trapping within the bulk heterojunction in the fixed light pulse width. In contrast, Figure 4b demonstrates a strongly duration-dependent EPSC enhancement under a fixed light intensity (0.22 mW/cm^2^) as the pulse width extended from 300 ms to 5000 ms. This arose from time-dependent carrier trapping dynamics: prolonged illumination allowed the sequential filling of multi-energy-level traps through synergistic interactions between CsPbBr_3_ QDs and PC_61_BM. Such pulse-width-dominated plasticity mimics biological spike-timing-dependent plasticity (STDP), which provides a key regulatory dimension for the construction of time-programmable neuromorphic devices.

PPF is one of the core manifestations of STP in biological synapses. It refers to the occurrence where two consecutive stimulation pulses applied to a synapse result in a significantly stronger postsynaptic response from the second pulse compared to the first. As illustrated in Figure 4c, the ternary heterojunction synaptic transistor successfully emulated this neurobiological phenomenon when subjected to two consecutive light pulses (450 nm; light intensity = 0.22 mW/cm^2^; pulse width = 100 ms; pulse interval = 1000 ms). The second EPSC peak (A_2_) was higher than the first EPSC peak (A_1_). As shown in Figure 4d, the ternary heterojunction synaptic transistor could effectively emulate PPF behavior, as described by following equation [33]:

(2) $$PPF=(I_{2}-I_{1})/I_{1}\times 100\%$$

where $I_{1}\;$and $I_{2}$ represent the currents recorded immediately after the first and the second light pulse stimulus, respectively. The experimental data were fitted using a double-exponential function [33]:

(3) $$y=yo+C_{1}exp(\frac{-t}{\tau_{1}})+C_{2}exp(\frac{-t}{\tau_{2}})$$

Here, t denotes the pulse interval, $C_{1}$ and $C_{2}$ correspond to the initial facilitation magnitudes, and $\tau_{1}$ and $\tau_{2}$ represent the characteristic relaxation times. The optimal fitting parameters for PPF behavior were determined as follows: $C_{1}$ = 4.9%; $C_{1}$ = 3.9%; $\tau_{1}$ = 0.53 s; and $\tau_{2}$ = 2.48 s. Notably, the relaxation times were consistent with the temporal characteristics observed in biological synaptic systems.

The learning process for novel information requires repetitive reinforcement through multiple cycles, analogous to how biological synapses strengthen their connectivity via frequent stimulation, resulting in enhanced postsynaptic currents with prolonged retention. To investigate the LTP of the perovskite QD-based ternary heterojunction synaptic transistor, we applied sequential light pulse stimuli (pulse width = 0.1 s; pulse interval = 0.15 s) with varying pulse numbers. As shown in Figure 4e, the EPSC amplitude of the last light pulse progressively amplified from 201 nA to 276 nA as the pulse number rose from 10 to 50. As illustrated in Figure 4f, the gain, defined as the ratio of the amplitude of the EPSC of the final pulse (A_n_) to that of the initial pulse (A_1_), demonstrated a progressive increase with an increasing pulse number. This cumulative potentiation arose from incremental charge trapping in the CsPbBr_3_ QDs and PC_61_BM by sequential light pulse stimulation. Following light pulse stimulus cessation, the postsynaptic current decayed due to accelerated carrier recombination. Crucially, higher light pulse numbers triggered a transition from STP to LTP, mimicking the synaptic consolidation process in hippocampal memory formation.

**Figure 5:**
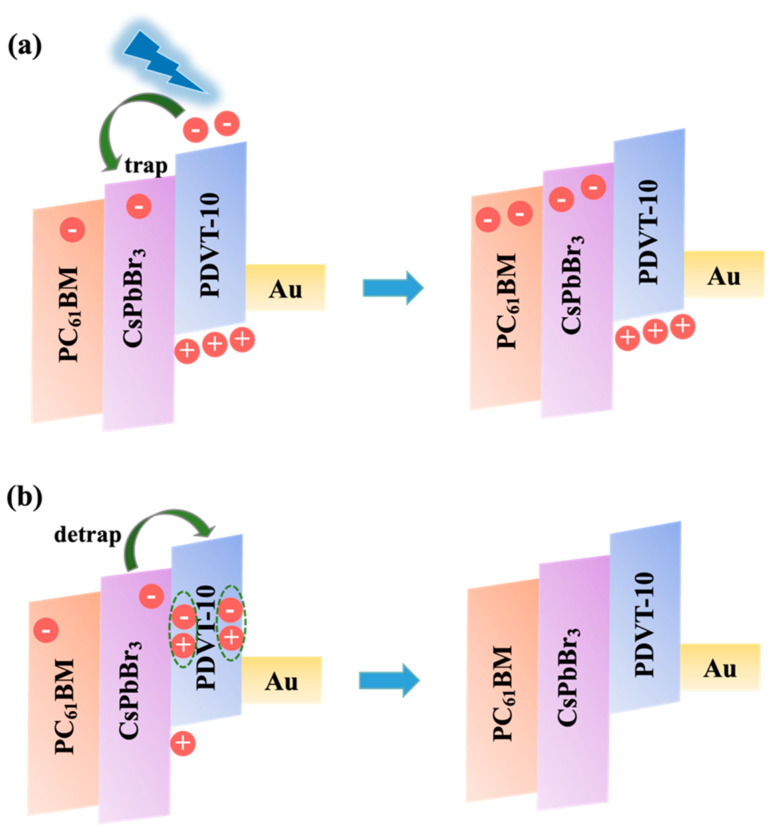
Operating mechanism of ternary heterojunction synaptic transistor. (**a**) Under light pulse. (**b**) After removal of light pulse.

The synaptic transistor device based on the ternary heterojunction PDVT-10–PC_61_BM–CsPbBr_3_ QDs developed in our study achieved the reversible modulation of channel conductivity through photogenerated carrier trapping dynamics. As illustrated in Figure 5, this regulation mechanism relied on the synergistic trapping effects of PC_61_BM acceptors and CsPbBr_3_ QDs. As shown in Figure 5a, under light pulse stimulation, the channel layer generated excitons through photon absorption. These excitons dissociated into free electrons and holes at the PDVT-10–PC_61_BM and PDVT-10–CsPbBr_3_ QD interfaces via energy barrier-driven separation. The electrons were subsequently captured by the PC_61_BM and CsPbBr_3_ QDs, leading to increased majority carrier (hole) concentration in the channel and enhanced conductivity. Figure 5b illustrates that upon the removal of light stimulation, the trapped electrons gradually escaped from the trapping centers through thermal release processes at the PDVT-10–PC_61_BM interface and PDVT-10–CsPbBr_3_ QD interface. These released electrons then recombined non-radiatively with holes in the channel, resulting in decreased hole concentration and progressive conductivity reduction. This dynamic process of carrier trapping and detrapping effectively mimicked the excitatory regulation mechanism of biological synapses. Notably, when subsequent light pulse stimulation was applied before complete carrier detrapping and recombination, newly generated excitons underwent repeated trapping processes. This cumulative trapping effect caused the continued elevation of hole concentration and conductivity enhancement, successfully emulating the synaptic weight potentiation characteristics observed in neural systems.

## 4. Conclusions

In summary, we successfully developed a ternary heterojunction synaptic transistor, which eliminates the need for complex functional layers while improving carrier capture capability. Compared to binary heterojunction synaptic transistors, the ternary heterojunction synaptic transistor exhibits an enlarged hysteresis window which is attributed to the synergistic trapping of acceptor materials and perovskite quantum dots. The memory window diminishes with increasing V_DS_ as accelerated carrier mobility reduces trapping probability. Meanwhile, the memory window increases with prolonged program/erase time, which enhances trapped charge density. Furthermore, the device successfully emulates typical synaptic functionalities, including EPSC, PPF, and transitions from STP to LTP. Moreover, the cumulative trapping mechanism under sequential light pulses enables synaptic weight potentiation, aligning with neural learning processes. This work demonstrates a facile fabrication approach for high-performance synaptic transistors, providing new insights for achieving neuromorphic computing and adaptive intelligent systems.

## Data Availability

The data that support the findings of this study are available from the corresponding author upon reasonable request.
